# Subsequent Ultrasound Vascular Targeting Therapy of Hepatocellular Carcinoma Improves the Treatment Efficacy

**DOI:** 10.3390/biology10020079

**Published:** 2021-01-22

**Authors:** Laith R. Sultan, Mrigendra B. Karmacharya, Stephen J. Hunt, Andrew K.W. Wood, Chandra M. Sehgal

**Affiliations:** 1 Ultrasound Research Laboratory, Department of Radiology, Perelman School of Medicine, University of Pennsylvania, 3620 Hamilton Walk, Philadelphia, PA 19104, USA; Mrigendra.Karmacharya@pennmedicine.upenn.edu (M.B.K.); Chandra.Sehgal@pennmedicine.upenn.edu (C.M.S.); 2 Penn Image-Guided Interventions Lab, Department of Radiology, Perelman School of Medicine, University of Pennsylvania, 421 Curie Blvd, 646 BRB II/III, Philadelphia, PA 19104, USA; Stephen.Hunt@pennmedicine.upenn.edu; 3 Department of Clinical Studies, School of Veterinary Medicine, University of Pennsylvania, 3800 Spruce Street, Philadelphia, PA 19104, USA; akwood@vet.upenn.edu

**Keywords:** hepatocellular carcinoma, cancer therapy, targeted therapy, tumor vasculature, contrast enhanced ultrasound, theranostics

## Abstract

**Simple Summary:**

The incidence of hepatocellular carcinoma (HCC) has consistently increased over the last two decades. Although surgical resection is the preferred treatment for HCC, approximately 80% of the patients are not suited for surgery because the tumors are in advanced stage of the disease and unresectable. Locoregional therapies like anti-vascular ultrasound (AVUS) provide a unique alternative for treatment. In this study we evaluate the use of repeated subsequent AVUS therapy as an approach to optimize the therapeutic efficacy by targeting tumor vasculature at different times. This approach can overcome the heterogeneity in tumor vasculature structure and functionality often observed at time of therapy. For this purpose, we evaluated two regiments of subsequent AVUS treatments with a predetermined time interval between the treatments. The results showed that AVUS when applied subsequently in a shorter duration, boosts the therapy effects showing more than 75% reduction in tumor perfusion post therapy. However, this improvement was lost for a longer duration between subsequent therapies. These findings demonstrate the significance of the viability of tumors for maximizing the therapeutic effects of tumor vasculature targeting therapies.

**Abstract:**

The response of hepatocellular carcinoma (HCC) to anti-vascular ultrasound therapy (AVUS) can be affected by the inherent differences in tumor vascular structure, and the functionality of tumor vessels at the time of treatment. In this study, we evaluate the hypothesis that repeated subsequent AVUS therapies are a possible approach to overcome these factors and improve the therapeutic efficacy of AVUS. HCC was induced in 30 Wistar rats by oral ingestion of diethylnitrosamine (DEN) for 12 weeks. A total of 24 rats received treatment with low intensity, 2.8 MHz ultrasound with an intravenous injection of microbubbles. Treated rats were divided into three groups: single therapy group (ST), 2-days subsequent therapy group (2DST), and 7-days subsequent therapy group (7DST). A sham control group did not receive ultrasound therapy. Tumor perfusion was measured by quantitative contrast-enhanced ultrasound (CEUS) nonlinear and power-Doppler imaging. Tumors were harvested for histologic evaluation of ultrasound-induced vascular changes. ANOVA was used to compare the percent change of perfusion parameters between the four treatment arms. HCC tumors treated with 2DST showed the largest reduction in tumor perfusion, with 75.3% reduction on average for all perfusion parameters. The ST group showed an average decrease in perfusion of 54.3%. The difference between the two groups was significant *p* < 0.001. The 7DST group showed a reduction in tumor perfusion of 45.3%, which was significant compared to the 2DST group (*p* < 0.001) but not different from the ST group (*p* = 0.2). The use of subsequent targeted AVUS therapies applied shortly (two days) after the first treatment enhanced the anti-vascular effect of ultrasound. This gain, however, was lost for a longer interval (1 week) between the therapies, possibly due to tumor necrosis and loss of tumor viability. These findings suggest that complex interplay between neovascularization and tumor viability plays a critical role in treatment and, therefore, must be actively monitored following treatment by CEUS for optimizing sequential treatment.

## 1. Introduction

Hepatocellular carcinoma (HCC) is the most common primary liver cancer, ranking 6th in incidence among other cancers worldwide [1]. According to WHO estimates, HCC represents the fourth most common cause of cancer related deaths around the globe, with more than 1 million mortalities expected by 2030 [2,3]. In the United States, the rate of death from liver cancer increased by 43% (from 7.2 to 10.3 deaths per 100,000) between 2000 and 2016 [4]. With a 5-year survival of 18%, liver cancer is the second most lethal tumor after pancreatic cancer.

Currently, HCC management is multidisciplinary and depends on tumor stage and liver function at the time of diagnosis and treatment. The standard of care is early resection and orthotopic liver transplant for those patients who are eligible for surgery [5,6,7]. Image-guided locoregional therapies (LRTs) are available for those patients in whom resection or transplantation is not a viable option, including ablation and transarterial chemoembolization (TACE) [8]. However, the invasive nature of these therapies remains a major concern for their use in clinical practice.

In our earlier studies, we introduced the use of antivascular ultrasound (AVUS) as a potential noninvasive locoregional therapy targeting the tumor vasculature [9,10,11,12,13]. Antivascular ultrasound (AVUS) therapy uses low-intensity unfocused ultrasound in combination with intravenously administered contrast agents (microbubbles) to disrupt the tumor vasculature. Microbubbles consist of a lipid, polymer, or protein shell and a gas core. Ultrasound stimulates the microbubbles to oscillate at their resonance frequency, damaging the adjacent endothelial cells and disrupting the tumor’s vascular supply and leading to ischemia and necrosis of neoplastic cells [14,15]. Because tumor vasculature is disorganized and tortuous, the blood flow is slower, leading to longer exposure of the tumor endothelium to the microbubbles compared to the surrounding healthy vasculature. 

One difficulty generally experienced with tumor vasculature targeting therapies is that susceptible tumor vasculature must be present and functional for the therapy to be effective [16,17,18]. These factors must be considered in optimizing the therapeutic effects of anti-vascular treatment. The heterogeneity in tumor vascular structure represents a possible barrier to a uniform response among tumors to AVUS therapy [16]. The continuous expression of VEGF-A (vascular endothelial growth factor A) at high levels over long periods during tumor growth mediates the development of exiting new blood vessels to more stable forms [17]. The mixture of “early immature” and “late mature” vessels present in tumors varies between tumors and their stage of growth. Accordingly, tumors may respond to treatment differently depending on the type of tumor vascular architecture. Another factor that can potentially affect the tumor response is that all the tumor blood vessels are not active all the time [18]. These factors are well recognized but are often not taken into consideration in therapeutic targeting of tumor vasculature. Since AVUS is a regional vascular therapy, the normal inactivity of some of the blood vessels at the time of treatment could potentially influence the response of the tumor to AVUS treatment. We hypothesized that the use of subsequent repeated therapies can help to overcome the limits of single treatment and thus provide a better control over tumor vascularity over time. Repetitive subsequent treatments target vessels at different times so that the tumors with initial incomplete response could be targeted in repeated treatments. Similar effects of subsequent repetitive therapies have been reported in clinical practice targeting of tumor vasculature by percutaneous ethanol injection (PEI) and TACE [19,20,21]. Repeated single sessions in PEI were shown to be more effective, with longer survival in patients with advanced HCC than those stratified to a single session [20]. Likewise, therapeutic improvement was observed with repeated sessions of TACE when given in a short duration between sessions [21]. 

The aim of this study is to evaluate the use of subsequent repeated treatments as a means of optimizing AVUS therapeutic effects. These effects are assessed using contrast-enhanced-ultrasound imaging (CEUS) in a diethylnitrosamine (DEN) induced orthotopic HCC model in rats, which is known to closely emulate the progression and growth of human HCC [22,23].

## 2. Methods

### 2.1. Animal Model

All animal protocols were approved by the university institutional animal care and use committee. Thirty Wistar male rats (Charles River Laboratories, Wilmington, MA, USA) weighing between 300 and 400 g, with an average of 6 months of age, were acquired and kept under controlled environmental conditions (25 °C and a 12 h light/dark cycle). After one week of acclimation, the rats were initiated on 0.01% diethylnitrosamine (DEN, Sigma Aldrich, St. Louis, MO, USA) in their drinking water, which they ingested ad libitum for 12 weeks. Ultrasound imaging and therapy were performed under anesthesia. The rat was placed in a non-rebreathing gaseous anesthetic system (VetEquip Inc, Livermore, CA, USA) and general anesthesia was induced with 4% isoflurane (IsoSol; VEDCO Inc, St Joseph, MO, USA). A nose cone was taped in place, anesthesia was maintained with 1% to 3% isoflurane and 1.5 to 2.0 L.min^−1^ oxygen, and a tail vein 26 G catheter (Covidien, Dublin, Ireland) was inserted. The animal was positioned supine on a heated bed, the abdomen was depilated, and continuous vital signs including body temperature, heart rate, and respiratory rate were recorded. At the completion of the study, the rat was euthanized by carbon dioxide asphyxiation, and necropsy was performed.

### 2.2. Antivascular Ultrasound Therapy

The rats were monitored for the development of tumors using grayscale ultrasound (21 MHz linear transducer, VisualSonics VevoLAZR, Fujifilm, Toronto, ON, Canada) beginning 11 weeks into the DEN diet and weekly thereafter. During monitoring sessions, diagnostic scans were performed, identifying and measuring visualized lesions. A single tumor most suitable for AVUS therapy was selected from the often-multiple tumors developed in the liver based on the tumor location (ventral and accessible to ultrasound imaging and therapy), size (6 to 14 mm diameter), hyperenhancement with microbubble administration, and minimal cystic or hemorrhagic changes as identified in the ultrasound images. After selecting and clearly identifying the tumor, the diagnostic transducer was secured in an adjustable mount to maintain a fixed position over the tumor. 

A low-intensity, continuous non-focused sinusoidal plane wave ultrasound therapy was performed at 2.8 MHz using 15 mm single element plane disk transducer. During therapy, the diagnostic transducer was replaced with the therapeutic probe in the same location above the tumor to keep the alignment with the imaged HCC. Therapy dose was selected based on our previous studies [9,10,11,12,13] Therapy consisted of sonication at 1.65 W/cm^2^ (corresponding to pressure amplitude of 0.23 MPa) and duty cycle of 100 percent, for a total of 6 min with 2 min on and off cycles. Then, 0.5 mL perflutren microbubbles (Definity, Lantheus, N. Billerica, MA, USA) were injected as a bolus via the tail vein catheter prior to the first insonation and 0.2 mL prior to the second 2 min insonation cycle; fractionating the microbubble administration ensured that microbubbles remained in circulation throughout the therapy.

The rats received ultrasound anti-vascular treatment from the 12th to the 17th week from the start of the DEN diet. The animals in the treatment group were divided into three groups: single therapy group (ST, *n* = 10 rats), 2-days subsequent therapy group (2DST, *n* = 7), and 7-days subsequent therapy group (7DST, *n* = 7). The single therapy group received one therapy session on a single day. The rats from this group were sacrificed on the same day for histology assessment. The 2DST group received a second therapy two days after the first therapy. The 7DST received the second treatment session 7 days after the first treatment. Two cases, one from the ST group and one from 7DST, did not respond to therapy due to technical difficulties in contrast injection and were not included in the analysis. The rats from subsequent therapy groups were sacrificed following the second treatment and the treated tumors were harvested for histologic analysis. A control group (*n* = 6) received a sham therapy in which the microbubbles were replaced with 0.7 mL normal saline, and the ultrasound therapy transducer was not switched on. The animals were sacrificed following sham treatment and the tumors were harvested for histologic analysis.

### 2.3. Pre- and Post-Therapy Contrast Enhanced Ultrasound Imaging of Tumor Perfusion

Contrast-enhanced ultrasound (CEUS) imaging was performed to assess tumor perfusion before and after AVUS treatment using nonlinear contrast (NLC) and power Doppler (PD) modes. This involved continuous serial imaging at 10 fps during the contrast enhancement of the tumor. Imaging presets and time compensation gain were optimized and kept fixed for pre- and post-therapy imaging. An intravenous bolus injection of 0.05 mL microbubbles was used for each imaging session.

Nonlinear contrast (NLC) images were analyzed by using the scanner software. The border of the tumor was manually outlined to define a region of interest (ROI) in the time series of contrast-enhanced images. The mean intensity of the ROI was fitted to a bolus perfusion model [24], and the following parameters were measured: peak enhancement (PE—the difference between the maximum amplitude and baseline intensity, which is proportional to microbubble concentration and indicative of relative blood volume); and perfusion index (PI—the area under curve divided by mean transit time of flow).

The contrast-enhanced PD images were analyzed using an IDL (Interactive Data Language)-based platform described earlier [22,23]. This involved measuring vascularity within the tumor region outlined on time series of power Doppler images. The color scale on the images was used to detect pixels with flow (colored pixels) and weighted according the strength of Doppler signal in the color bar. The number of colored pixels, the mean blood volume through each colored pixel and the total number of pixels enclosed within each region were measured as a function of time during contrast enhancement. Fractional tumor area covered by colored pixels at the peak enhancement was used to determine the peak vascular area (VAR). Flow volume perfusion index (FVPI) was measured by the ratio of the area under the enhancement-time curve and the mean transit time. 

The NLC and Doppler measurements were made on the whole tumor as well as regionally in the center and periphery of the tumor.

### 2.4. Statistical Analysis

The average (±standard error SE) of the quantitative data for the perfusion parameters (PE, PI, VAR, FVPI) of the treatment groups was compared by one-tailed paired Student’s *t*-tests on the decrease in tumor perfusion after therapy. *p* < 0.05 was considered significant. One-way analysis of variance (ANOVA) was used to compare the percent change of perfusion parameters between the four treatment arms. ANOVA was followed by Tukey’s test as a post hoc test. D’Agostino-Pearson test was used to check the normal distribution of the data. Statistical analysis was performed using MedCalc (version 19.0.5, MedCalc Software Ltd., Ostend, Belgium). 

### 2.5. Histology Assessment

At the completion of each study, tumor samples were collected for histological examinations. Tumors were preserved in 10% phosphate-buffered formalin for 48–72 h before being transferred to 50% ethanol, embedded in paraffin, and processed for histological examination with hematoxylin and eosin (H&E) staining. The tumor slices were examined under microscope (Zeiss Microscope Axio Imager Z1, White Plains, NY, USA). Images were acquired and the percentage area of hemorrhage was recorded using ImageJ software [25]. The acquired H&E-stained images of the whole tumors were deconvoluted and threshold adjusted in ImageJ. The percentage area of the red color was measured and average value (+/− SEM) was presented for each group.

## 3. Results

### 3.1. Animal Model

**Figure 1:**
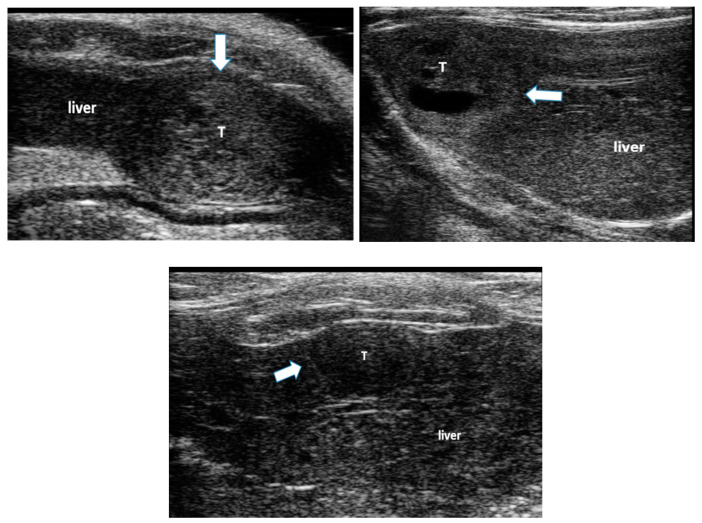
Examples of hepatocellular carcinoma (HCC) tumors developed in the liver of rats following ingestion of diethylnitroseamine (DEN) for 12 weeks. The white arrows point to HCC tumors (T) surrounded by liver tissue. Liver tissue is hyper-echoic and coarse due to the cirrhotic changes.

All animals developed liver disease and multiple foci of hepatocellular carcinoma (HCC), seen on B-mode ultrasound imaging (Figure 1). These tumors were associated with liver changes including increased echogenicity and coarseness of the image texture, indicating liver fibrosis and cirrhosis [22,26], ascites and hepatosplenomegaly. 

### 3.2. The Effects of Single AVUS Therapy of HCC Tumors

HCC tumors that received a single treatment showed a reduction in NLC derived perfusion parameters, where PE decreased from 6.2 ± 2.4 to 3.0 ± 1.6 post therapy, *p* = 0.06, with a 51.4% reduction in tumor perfusion. PI, on average, dropped from baseline 6.4 ± 2.3 to 2.9 ± 1.4, *p* = 0.05, with a percentage decrease of 57.0%. Similarly, power Doppler parameters showed a reduction in tumor perfusion after AVUS therapy, where VAR decreased from 63.1 ± 11.2 to 31.0 ± 11.4, *p* = 0.08, with a percentage decrease of 54.5%. FVPI decreased from 37.35 ± 5.99 to 17.67 ± 5.52, *p* = 0.03, representing a decrease of 58.3%.

**Figure 2:**
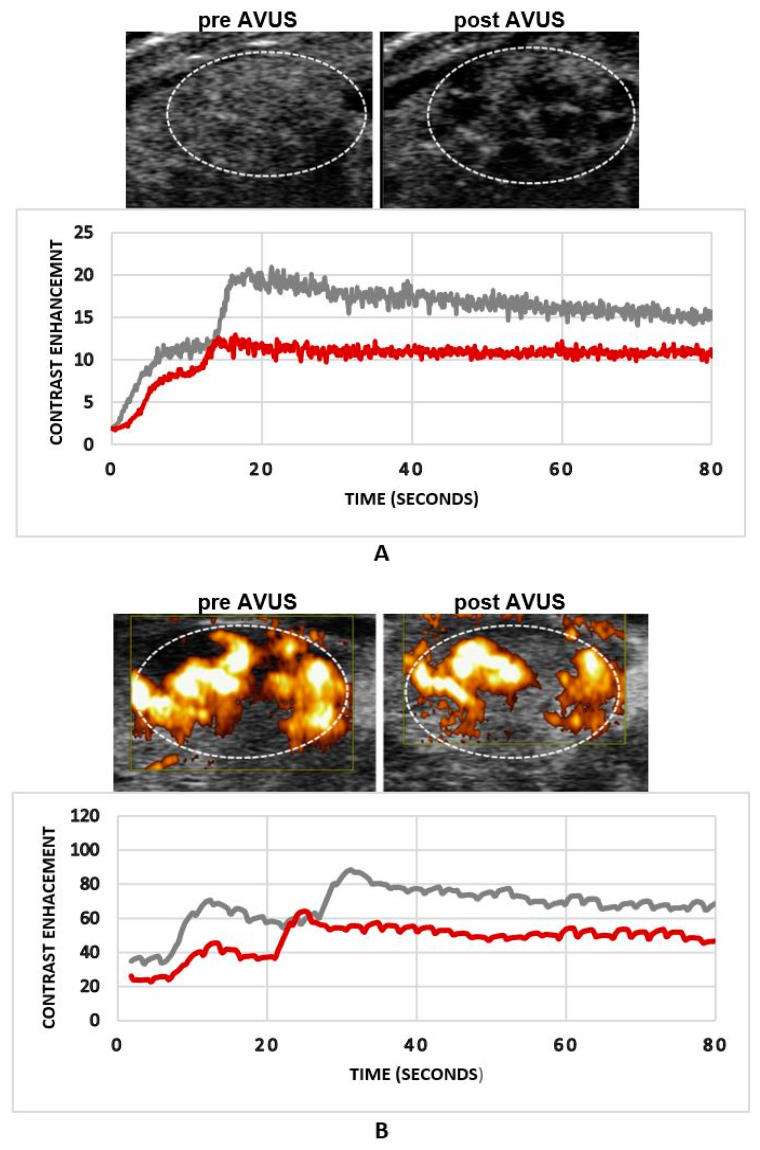
An example of an HCC tumor that received a single anti-vascular ultrasound therapy (AVUS) therapy. Panel (**A**) shows the reduction in tumor perfusion post therapy on nonlinear contrast (NLC) imaging. The tumor image post therapy shows multiple dark hypoechoic localized areas indicating lesser contrast coming after therapy. The dilution curve shows that tumor contrast enhancement was decreased more than 40% post therapy. Panel (**B**) shows the changes in tumor perfusion after therapy on power Doppler imaging. It is notable that the changes are more evident on NLC imaging compared to power Doppler (PD).

Figure 2 shows an example of an HCC tumor that received a single AVUS therapy on NLC and PD imaging modes. Both modes showed a decrease in tumor perfusion after therapy and the reduction in tumor perfusion was more evident on NLC images (Figure 2A) than on power Doppler (Figure 2B). Multiple localized hypoechoic areas indicating lesser inflow of contrast agent to these areas were observed post-therapy (Figure 2A). The dilution curve showed a reduction in tumor contrast enhancement of about 50% post therapy by NLC, compared to 30% by PD.

### 3.3. The Effects of Subsequent AVUS Therapies within Two Days (2DST Group)

The HCC tumors that were treated subsequently within two days apart showed a remarkable reduction in tumor perfusion after therapy, where PE decreased from 5.4 ± 2.2 to 1.1 ± 0.4, *p* = 0.02, after therapy, with a percentage decrease of 82.7%. Mean PI showed a similar significant decrease of 82.6%, from 6.0 ± 3.5 a.u. to 1.4 ± 0.6 a.u., *p* = 0.03. 

Likewise, power Doppler parameters showed a significant reduction in perfusion kinetics after AVUS therapy, where VAR decreased from 59.8 ± 12.8 a.u. to 20.6 ± 8.5 a.u., *p* = 0.02, with a 65.5% decrease, and FVPI decreased from 31.37 ± 7.78 to 8.32 ± 1.39, 70.35, *p* = 0.004.

**Figure 3:**
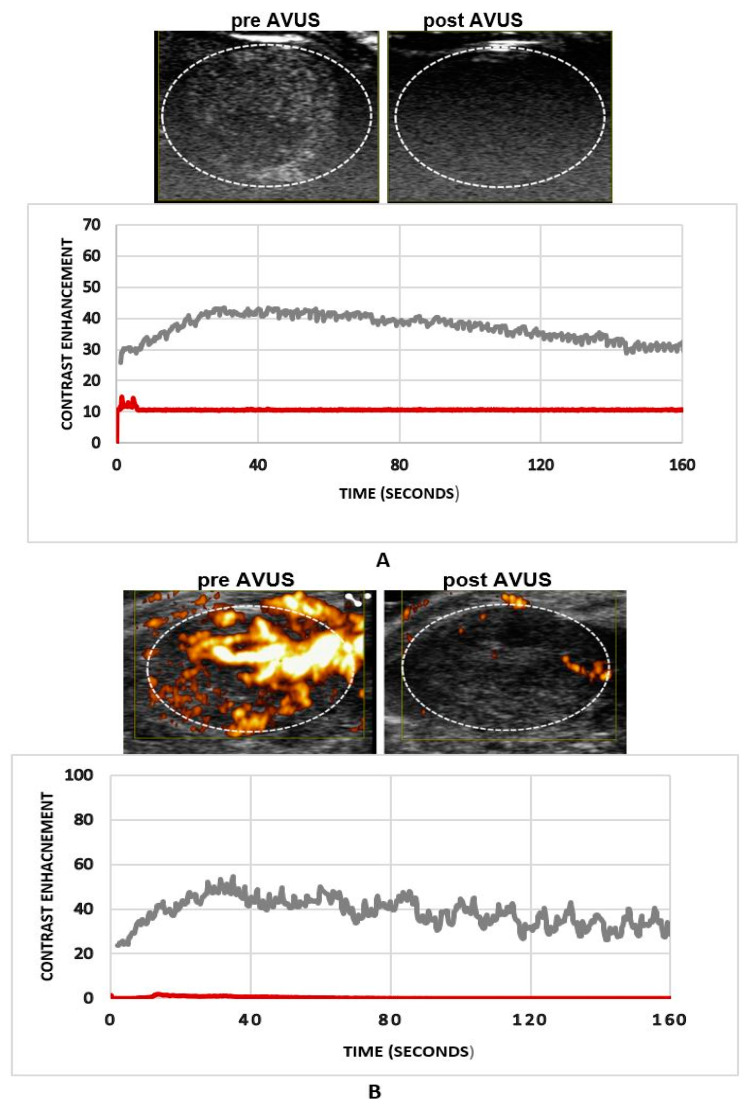
An example of an HCC tumor that received subsequent therapies within two days. Both NLC and PD images (panel (**A**) and (**B**)) show a significant reduction (more than 90%) in tumor perfusion after therapy seen in this case. Minimal contrast flow seen in the treated tumor post therapy.

Figure 3 shows an example of an HCC tumor that received subsequent therapies within two days. A marked reduction in tumor perfusion was observed on both NLC (Figure 3A) and PD (Figure 3B) images. The dilution curve derived from both modes demonstrated that tumor perfusion decreased more than 95% for this case, where very minimal contrast flow is seen after therapy.

### 3.4. The Effects of Subsequent AVUS Therapies within 7 Days on HCC Tumors (7DST Group)

**Figure 4:**
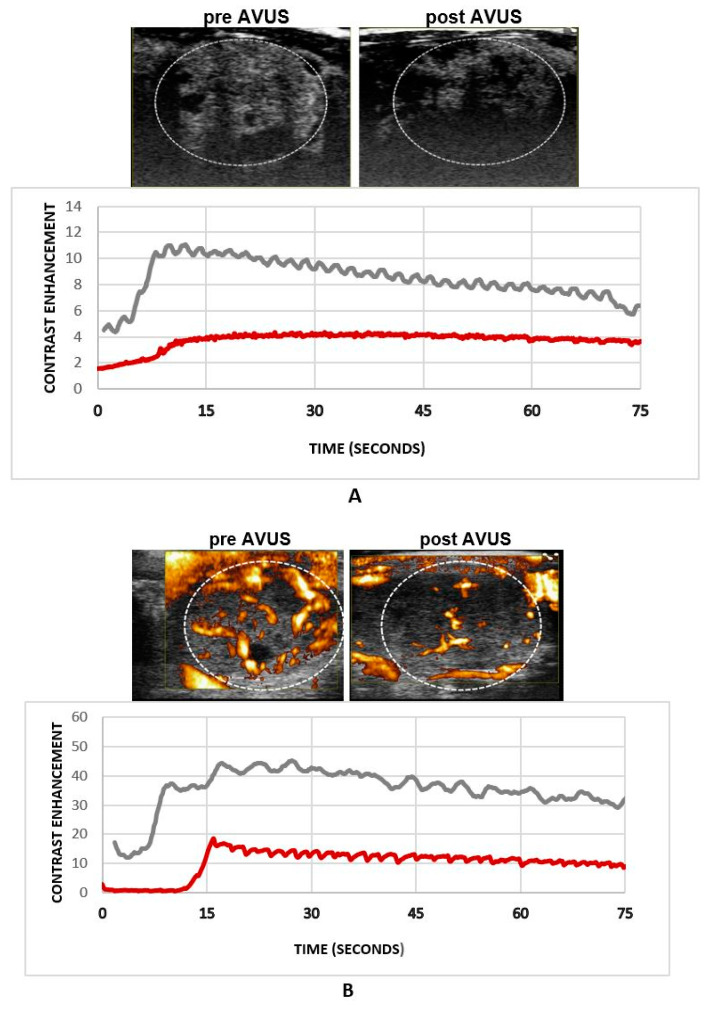
An example of an HCC tumor that received subsequent therapies within two days. Both panels (**A**) and (**B**) demonstrated a reduction in tumor perfusion after therapy in this case.

HCC tumor perfusion decreased after subsequent therapies applied within 1 week, where mean PE went down from a baseline of 3.4 ± 1.1 to 1.6 ± 1.3 post therapy, *p* = 0.2, showing a 52.6% decrease in tumor vascularity. PI decreased from 2.9 ± 1.0 to 1.6 ± 1.1, *p* = 0.3, with a 47.2% decrease in tumor perfusion. Similarly, PD derived VAR decreased from 58.8 ± 8.8 to 33.7 ± 2.9, 49.6%, *p* = 0.006, and FVPI went down from 21.63 ± 4.66 to 14.72 ± 1.16, *p* = 0.07, with 32.0%. Figure 4 shows an example of an HCC tumor that received subsequent therapies within a week, where tumor perfusion decreased after therapy as shown by NLC (Figure 4A) and PD (Figure 4B) images.

### 3.5. Comparison of the Effects for Single vs. Subsequent AVUS Therapies on HCC Tumors 

**Figure 5:**
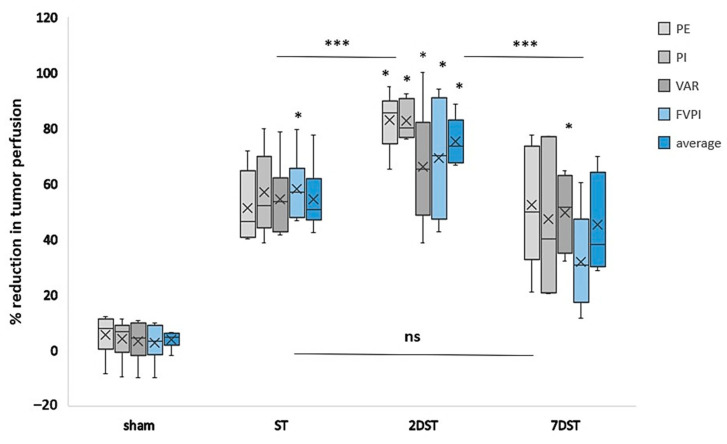
Box plot comparing the percentage reduction in tumor perfusion of single therapy (ST), subsequent therapies within two days (2DST), subsequent therapies with 7 days (7DST) and sham groups. Clearly, the 2DST group outperforms the other groups, showing a larger percentage decrease in tumor perfusion in all perfusion parameters studied. Sham therapy showed no or minimal changes. PE; peak enhancement, PI; perfusion index, VAR; vascularity area, FVPI; flow volume perfusion index, and, average represents the average of all parameters. * refers to significant percentage reduction (*p* < 0.05). *** refers to significant difference between groups (*p* < 0.001), and (ns) refers to non-significant difference between groups.

**Figure 6:**
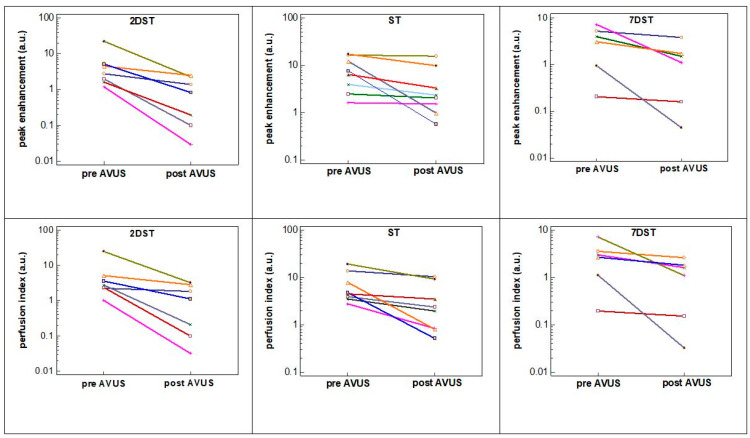
The change in tumor perfusion parameters pre- and post-therapy compared for individual cases in the 2DST, ST and 7DST groups. HCC tumors in the 2DST group showed more uniformity in reduction in tumor perfusion after therapy compared to the other two groups. The *x*-axis represents the therapy time points (pre- and post-AVUS therapy).

The effects of ST, 2DST, 7DST and sham therapies on HCC tumor perfusion are compared in Figure 5. In the figure, it is notable that the tumors that received subsequent therapies within two days (2DST) showed the largest percentage reduction in tumor perfusion following therapy, with an average change for all parameters of 75.3% compared to lesser effects seen in tumors that received a single therapy (ST) (54.5%). The difference between the two groups was significant (*p* < 0.001). The reduction in tumor perfusion was significantly larger in 2DST compared to 7DST, which shows a reduction in tumor perfusion of 45.3%, *p* < 0.001. The comparison of ST and 7DST did not show a significant difference between the two groups. On a case-by-case basis, the decrease in tumor perfusion was more uniform in the 2DST group, compared to the ST or 7DST groups (Figure 6). 

### 3.6. Tumor Histopathology Shows Differences in Effects of Single vs. Subsequent AVUS Treatments 

**Figure 7:**
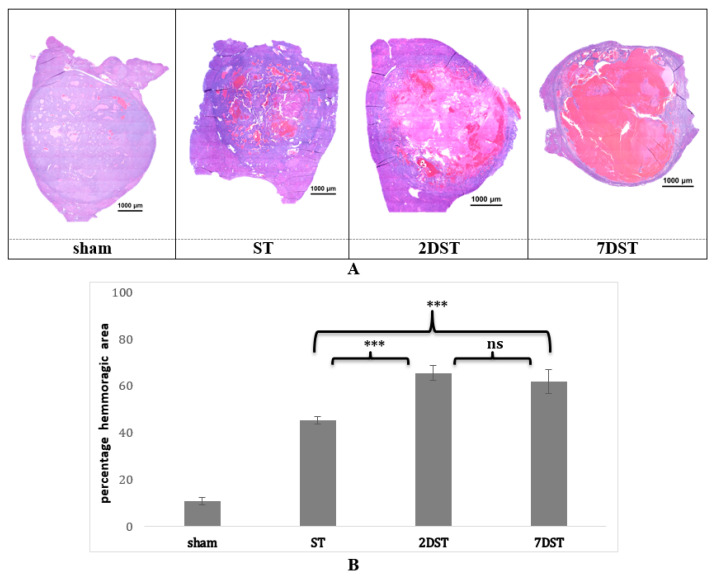
(**A**) Hematoxylin and eosin (H&E) staining of the HCC tumor samples. Small hemorrhagic pools are seen in the tumors treated with a single (ST) compared to larger pools seen in subsequent (2DST, 7DST) therapies. More necrotic tissue is seen in 7DST tumors. No sensible change observed in sham cases. Area enclosed within the broken line represents the tumor. (**B**) Quantitative analysis of the hemorrhagic area of the HCC tumor samples. The bars represent the percentage of hemorrhagic area in the sham tumors, or treated with sham, single (ST) or subsequent in two days (2DST) or subsequent in seven days (7DST). The difference in the percentage of hemorrhagic area between sham vs. single Rx (*p* < 0.001), sham vs. 2DST and 7DST (*p* < 0.001), and ST vs. 2DST and 7DST (*p* < 0.001) were statistically significant. *** represent significant difference between groups, while ns means non-significant difference.

Histological analysis of the H&E-stained HCC tumors demonstrated that percentage hemorrhagic areas significantly increased after subsequent AVUS therapies (Figure 7A). H&E-stained HCC tumors showed a marked increase in hemorrhagic pools in treated tumors compared to the sham group. The hemorrhagic area in the 2DST group was much greater than that in the ST group. The hemorrhagic areas in both 2DST and 7DST were comparable, but the necrotic area was larger in 7DST compared to 2DST. Quantitatively, hemorrhagic areas in sham, ST, 2 DST, and 7 DST were 10.8 ± 1.4%, 45.4 ± 1.5%, 65.5 ± 2.2%, and 61.8 ± 1.8%, respectively (Figure 7B). The differences in the percentage of hemorrhagic area between sham vs. ST (*p* < 0.001), sham vs. 2DST and 7DST (*p* < 0.001), and ST vs. 2DST and 7DST (*p* < 0.001) were statistically significant. However, the difference between 2DST and 7DST was not significant (*p* = 0.2). 

### 3.7. AVUS Therapy Induced Changes with Time Following Treatment

**Figure 8:**
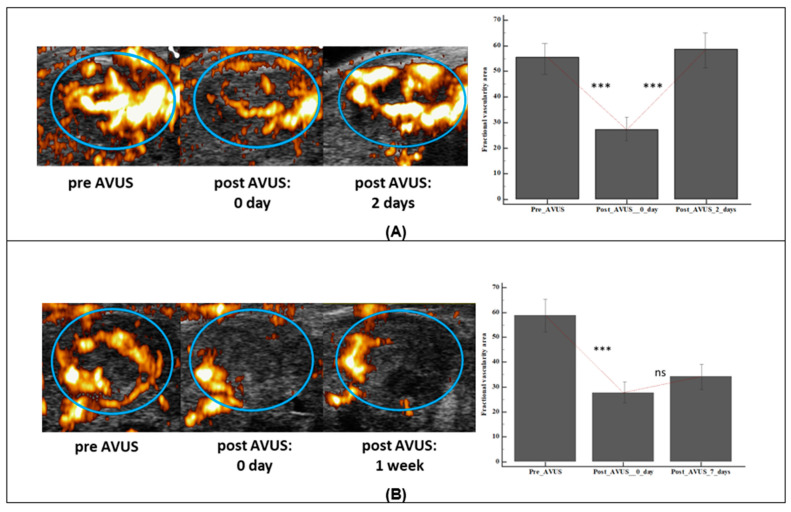
The changes in HCC tumor vascularity with time after 1st AVUS therapy in 2DST and 7DST groups. The upper panel (**A**) shows an example HCC tumor from the 2DST group that showed a reduction in vascularity immediately after therapy, and when followed two days later, tumor vascularity increased again to same level of baseline. The bar graph on the side shows the change in the vascularity area (VAR) for all 2DST tumors after first therapy. In the lower panel (**B**) an example of an HCC tumor that was followed a week later after therapy. No gain was observed in vascularity level from post therapy one week later. *** represent significant difference, while ns means not significant.

In the 2DST group, the vascularity of treated tumors returned to its baseline level two days following the first therapy, while the 7DST cases that were followed 7 days post first therapy did not show the same trend (Figure 8). In 2DST, tumor vascularity decreased after the first therapy from a mean vascularity area of 59.8 ± 12.8 to 27.2 ± 10.3. Two days later, tumor vascularity returned to 58.6 ± 13.3. In contrast to the 2DST group, the tumor vascularity continued to decrease following the first treatment in the 7DST group. Mean fractional vascularity area at baseline decreased from 58.8 ± 8.8 to 27.7 ± 6.6 after the first treatment and remained attenuated at 34.2 ± 3.3 on the seventh day post-treatment. 

### 3.8. Regional Vascularity Differences in Tumor Response to AVUS Therapy

In both groups that received subsequent therapies, viz., 2DST and 7DST groups, it was observed that following the first treatment, the tumor vascularity decreased largely in the rim region. The vascularity decreased by 77.8% and 74.1% in the rim compared to 52.6% and 48.6% in the center of the 2DST and 7DST groups, respectively. The reduction in the vascularity of the two regions was significant (*p* < 0.05) for both the groups. 

## 4. Discussion

In recent years, therapies targeting tumor vasculature are playing an increasing role in cancer treatment, particularly in HCC [27,28]. The selective targeting of established tumor vasculature represents an attractive new anticancer strategy, distinct from inhibiting angiogenesis [29]. This is based on the concept that, in contrast to targeting individual tumor cells, the killing of relatively few vascular endothelial cells could result in the death of a large area of the tumor from a lack of oxygen and nutrients. Additionally, cells that comprise vasculature, such as endothelial cells, are genetically more stable than tumor cells and less likely to acquire changes similar to those causing drug resistance [29]. However, for anti-vascular therapies to be effective, the targeted tumor must be highly vascular and functional. Generally, tumor vessels differ from normal vasculature because tumor vasculature has irregular caliber and is leaky and tortuous with sluggish blood flow when compared with normal vessels. Even within the same tumor, its vasculature is composed of vessels that differ from each other, and this heterogeneity has therapeutic implications when tumor vessels are targeted. These inherent differences in the vasculature of the tumors, both morphologic and functional, at different times could influence their response to therapy. In this study, we evaluated the use of subsequent treatments of AVUS therapy as an approach to overcome the issues related to the inherent differences in tumor vascular structure and functionality in order to optimize the therapy effects. 

### 4.1. The Use of Subsequent Repeated Treatments Improves AVUS Therapeutic Effects

HCC tumors that were given subsequent therapies within a short duration (two days apart; 2DST) showed a substantially large reduction in tumor perfusion of more than 75% on average, which was significantly larger than that seen in tumors which received a single therapy, showing about 54% reduction only. Several factors could be responsible for the observed difference between the two groups. First, the use of subsequent treatments provides the opportunity of targeting new blood vessels formed by angiogenesis between the treatments. Second, the damages to the outer vessels in the first treatment allow an increased penetration of ultrasound beam to the center of the tumor in following treatments. Our results showed that the rim region of the tumor was highly affected by AVUS and demonstrated a larger reduction in tumor vascularity following the first treatment, compared to lesser effects observed in the center region. An additional explanation for the increased effectiveness of subsequent therapies is related to the activity of the microvasculature, which varies with time [18]. Earlier studies showed that a variable proportion of tumor vessels are at times nonfunctional and have no blood flow. The repeated treatments allow for the targeting of blood vessels that were not active in the initial treatment. The imaging findings that showed the 2DST group to have larger vascular damage compared to the single therapy group were confirmed by histopathology, where larger hemorrhage areas were observed in the 2DST group compared to the single therapy group.

### 4.2. Subsequent Therapies within a Short Time Interval Outperform Subsequent Therapies Given within Longer Duration

This study also investigated the effect of time interval between subsequent therapies on tumor response. The selection of the two time points for subsequent therapies at 2 and 7 days was based on our aim to compare the effects of subsequent AVUS therapies when given in a short interval and a longer interval. Stronger treatment effects were observed in the group that received subsequent therapies in two days, compared to a much lesser effect in the group that received the second treatment a week later. This was rather an expected finding as the immediate changes could be boosted with a second therapy in a short duration, while waiting for a longer duration till the effects of the first therapy have been diminished would have lesser effects. The stronger effects of a subsequent treatment within a shorter duration could be related to the tumor being more viable two days from therapy and in an earlier stage of tumor growth and ongoing angiogenesis. On the other hand, seven days later, changes related to tumor aging occur, including tumor necrosis, hemorrhage, calcification and loss of vascularity, making them less responsive to therapy. The histopathology analysis showed more necrosis in 7DST compared to 2DST, reflecting the tumor aging process.

An interesting observation in the subsequent therapy tumor groups is that, following the first AVUS treatment, tumor perfusion increased to the baseline level over the first two days, while it did not show the same pattern when evaluated after a week. These findings also indicate the viability of tumors within two days from the first therapy, as angiogenesis and the formation of new vessels make them ideal for a second therapy. On the other hand, seven days later, the tumor ages and loses some of its vascularity, making it less responsive to a subsequent therapy.

### 4.3. Limitations 

This study is not without limitations. An important concern is whether there are any unwanted effects with multiple treatments. AVUS therapy is ideally suited for recurring treatments due to the use of low energy ultrasound, which allows subsequent treatments. Although all the animals tolerated the multiple treatments well, a more detailed study investigating the side effects of such treatment is needed. 

## 5. Conclusions

The use of tumor vascular targeting therapies remains challenging and can be affected by the inherent differences in the structure and functionality of HCC tumor vasculature. To overcome these limitations, we evaluated two predetermined regiments of subsequent AVUS treatments with a fixed time interval between the treatments. The results showed that AVUS therapy effects were boosted when applied subsequently in a shorter duration. This improvement, however, was lost for a longer duration between subsequent therapies. These findings demonstrate the significance of the viability of tumors for targeting the tumor vasculature and maximizing the therapeutic effects. With improved imaging techniques such as contrast enhanced endoscopic ultrasound (CEU) [30], with its inherent ability to detect smaller lesions, future developments of our proposed therapy (AVUS) will involve using CEU to assess the treatment effects in non-accessible parts with traditional imaging modalities.

## Data Availability

The data presented in this study are available on request from the corresponding author. The data are not publicly available due to the evolving nature of the project.

## References

[1] International Agency for Research on Cancer (2019). Cancer Tomorrow.

[2] WHO (2018). Projections of Mortality and Causes of Death, 2016 to 2060.

[3] Forner A., Reig M., Bruix J. (2018). Hepatocellular carcinoma. Lancet.

[4] Xu J. (2018). Trends in Liver Cancer Mortality among Adults Aged 25 and Over in the United States, 2000–2016. NCHS Data Brief.

[5] Finn R.S., Qin S., Ikeda M., Galle P.R., Ducreux M., Kim T.-Y., Kudo M., Breder V., Merle P., Kaseb A.O. (2020). Atezolizumab plus Bevacizumab in Unresectable Hepatocellular Carcinoma. N. Engl. J. Med..

[6] Bruix J., Sherman M. (2011). Management of hepatocellular carcinoma: An update. Hepatology.

[7] Hanahan D., Weinberg R.A. (2011). Hallmarks of cancer: The next generation. Cell.

[8] Dendy M.S., Ludwig J.M., Stein S.M., Kim H.S. (2019). Locoregional Therapy, Immunotherapy and the Combination in Hepatocellular Carcinoma: Future Directions. Liver Cancer.

[9] D’Souza J.C., Sultan L.R., Hunt S.J., Gade T.P., Karmacharya M.B., Schultz S.M., Brice A.K., Wood A.K.W., Sehgal C.M. (2019). Microbubble enhanced ultrasound for the antivascular treatment and monitoring of hepatocellular carcinoma. Nanotheranostics.

[10] Hunt S.J., Gade T., Soulen M.C., Pickup S., Sehgal C.M. (2015). Antivascular Ultrasound Therapy. J. Ultrasound Med..

[11] Levenback B.J., Sehgal C.M., Wood A.K.W. (2012). Modeling of thermal effects in antivascular ultrasound therapy. J. Acoust. Soc. Am..

[12] Wood A.K.W., Ansaloni S., Ziemer L.S., Lee W.M.-F., Feldman M.D., Sehgal C.M. (2005). The antivascular action of physiotherapy ultrasound on murine tumors. Ultrasound Med. Biol..

[13] Wood A.K., Bunte R.M., Price H.E., Deitz M.S., Tsai J.H., Lee W.M.-F., Sehgal C.M. (2008). The Disruption of Murine Tumor Neovasculature by Low-intensity Ultrasound—Comparison Between 1- and 3-MHz Sonication Frequencies. Acad. Radiol..

[14] Presset A., Bonneau C., Kazuyoshi S., Nadal-Desbarats L., Mitsuyoshi T., Bouakaz A., Kudo N., Escoffre J.M., Sasaki N. (2020). Endothelial Cells, First Target of Drug Delivery Using Microbubble-Assisted Ultrasound. Ultrasound Med. Biol..

[15] Kooiman K., Roovers S., Langeveld S.A., Kleven R.T., Dewitte H., O’Reilly M.A., Escoffre J.-M., Bouakaz A., Verweij M.D., Hynynen K. (2020). Ultrasound-Responsive Cavitation Nuclei for Therapy and Drug Delivery. Ultrasound Med. Biol..

[16] Nagy J.A., Dvorak H.F. (2012). Heterogeneity of the tumor vasculature: The need for new tumor blood vessel type-specific targets. Clin. Exp. Metastasis.

[17] Zhu W., Kato Y., Artemov D. (2014). Heterogeneity of Tumor Vasculature and Antiangiogenic Intervention: Insights from MR Angiography and DCE-MRI. PLoS ONE.

[18] Fisher D.T., Muhitch J.B., Kim M., Doyen K.C., Bogner P.N., Evans S.S., Skitzki J.J. (2016). Intraoperative intravital microscopy permits the study of human tumour vessels. Nat. Commun..

[19] Iavarone M., Colombo M. (2013). Management of Hepatocellular Carcinoma. Viral Hepatitis.

[20] Dettmer A., Kirchhoff T., Gebel M., Zender L., Malek N.P., Panning B., Chavan A., Rosenthal H., Kubicka S., Krusche S. (2006). Combination of repeated single-session percutaneous ethanol injection and transarterial chemoembolisation compared to repeated single-session percutaneous ethanol injection in patients with non-resectable hepatocellular carcinoma. World J. Gastroenterol..

[21] Liu C.-Y., Chen K.-F., Chen P. (2015). Treatment of Liver Cancer. Cold Spring Harb. Perspect. Med..

[22] D’Souza J.C., Sultan L.R., Hunt S.J., Schultz S.M., Brice A.K., Wood A.K.W., Sehgal C.M. (2019). B-mode ultrasound for the assessment of hepatic fibrosis: A quantitative multiparametric analysis for a radiomics approach. Sci. Rep..

[23] Karmacharya M.B., Sultan L.R., Kirkham B.M., Brice A.K., Wood A.K., Sehgal C.M. (2020). Photoacoustic Imaging for Assessing Tissue Oxygenation Changes in Rat Hepatic Fibrosis. Diagnostics.

[24] Needles A., Arditi M., Rognin N., Mehi J., Coulthard T., Bilan-Tracey C., Gaud E., Frinking P., Hirson D., Foster F. (2010). Nonlinear Contrast Imaging with an Array-Based Micro-Ultrasound System. Ultrasound Med. Biol..

[25] Schneider C.A., Rasband W.S., Eliceiri K.W. (2012). NIH Image to ImageJ: 25 years of image analysis. Nat. Methods.

[26] Jin N., Deng J., Chadashvili T., Zhang Y., Guo Y., Zhang Z., Yang G.-Y., Omary R.A., Larson A.C. (2010). Carbogen Gas–Challenge BOLD MR Imaging in a Rat Model of Diethylnitrosamine-induced Liver Fibrosis. Radiology.

[27] Thorpe P.E. (2004). Vascular Targeting Agents as Cancer Therapeutics. Clin. Cancer Res..

[28] Korpanty G., Brekken R.A. (2011). Update on vascular disrupting agents for cancer therapy. Therapy.

[29] Brekken R.A., Li C., Kumar S. (2002). Strategies for vascular targeting in tumors. Int. J. Cancer.

[30] Girotra M., Soota K., Dhaliwal A.S., Abraham R.R., Garcia-Saenz-De-Sicilia M., Tharian B. (2018). Utility of endoscopic ultrasound and endoscopy in diagnosis and management of hepatocellular carcinoma and its complications: What does endoscopic ultrasonography offer above and beyond conventional cross-sectional imaging?. World J. Gastrointest. Endosc..

